# Friction and Wear Behaviors of PI/MoS_2_ Bearing Cage Composite Under Elevated Temperature Conditions

**DOI:** 10.3390/polym18060716

**Published:** 2026-03-16

**Authors:** Qichang Guo, Chuanbo Liu, Juncheng Lv, Shutian Liu

**Affiliations:** 1 School of Mechanical and Electronic Engineering, Wuhan University of Technology, Wuhan 430070, China; 2 SAIC GM Wuling Automobile Company (SGMW), Liuzhou 545027, China

**Keywords:** polyimide, molybdenum disulfide, high temperature, friction and wear, bearing cage

## Abstract

The drive systems of new energy vehicles, which employ high-speed motors and low-viscosity lubricants, often subject motor bearings to high-temperature and oil-starved conditions. This can lead to the deformation of polymer bearing cages, resulting in abnormal vibration and noise. In this study, polyimide/molybdenum disulfide (PI/MoS_2_) composites were prepared, and their thermal stability was characterized using a dynamic mechanical analysis (DMA). High-temperature friction and wear tests against ceramic balls were conducted on a multifunctional tribometer. The wear behavior and surface element distribution were examined by laser confocal microscopy (LCSM), scanning electron microscopy (SEM), and energy-dispersive spectroscopy (EDS). Results indicate that the PI-MoS_2_ composites effectively mitigate surface contact deformation with rising temperatures, reducing the wear loss by around 30% compared to pure PI. This improvement is attributed to the enhanced heat resistance from MoS_2_ and the formation of a lubricating film during friction. The findings provide guidance for selecting and designing composite materials for high-speed bearing cages.

## 1. Introduction

The development and promotion of electric vehicles (EVs) represents a crucial strategy for mitigating air pollution, alleviating environmental pressures, and addressing the global energy crisis. It also drives the transformation of the traditional automotive industry toward the ‘new four modernizations’, which are electrification, connectivity, intelligence, and sharing [1,2]. Electrification is at the forefront of this transition, with the dedicated hybrid transmission (DHT) serving as a core component. The DHT operates essentially through an electric drive, wherein the motor acts as a new power source alongside the internal combustion engine [3]. However, the application of high-speed motors and low-viscosity lubricants in EVs often exposes drive motor bearings within DHT systems to harsh operating conditions characterized by high temperatures and starved lubrication. These adverse conditions lead to the deformation of the polymer bearing cage, resulting in abnormal vibration and noise and, in severe cases, causing premature bearing failure [4,5,6].

The physical, chemical, and thermal properties of the bearing cage material, along with its lubrication performance, are vital for reliable DHT operation. Notably, approximately 60% of bearing cage failures are attributed to inadequate lubrication. For instance, when the water content in the bearing grease exceeds 0.5%, the wear rate of the cage material can increase threefold [7]. Furthermore, the pursuit of higher transmission efficiency and improved heat dissipation in DHT systems has led to the adoption of low-viscosity lubricants to reduce churning losses [8]. However, this reduction in viscosity increases the risk of hardware failure within the electric drive system [9]. The resulting oil-starved condition intensifies the contact wear between the bearing balls and the cage, potentially leading to ‘equatorial’ damage on the balls and, in severe cases, abnormal vibration and noise [10].

As the power density of high-speed motors continues to rise, drive motors in DHT systems frequently operate at speeds exceeding 10,000 revolutions per minute (rpm), raising bearing temperatures to approximately 120 °C [11]. Moreover, to mitigate electrical discharge damage, ceramic ball bearings are increasingly replacing traditional steel bearings [12]. The relatively poorer thermal conductivity of ceramic materials can further elevate operating temperatures to around 150 °C [13]. Common cage materials, such as nylon, polytetrafluoroethylene, and polyimide-based polymer composites, are susceptible to deformation and accelerated wear under sustained high temperatures, thereby shortening the bearing service life [14].

This study focuses on polyimide (PI) composite cage materials, employing molybdenum disulfide (MoS_2_) as a solid lubricant to fabricate PI/MoS_2_ composites via physical modification. Investigating the influence of thermomechanical properties and thermal stability on the high-temperature friction and wear behavior of these composites is significant. This work aims to elucidate the mechanisms of contact deformation and wear formation under high-temperature conditions, providing valuable insights for the development of self-lubricating, low-friction, low-wear, and high-heat-resistance composite materials for bearing cages.

## 2. Methods and Experiments

### 2.1. Testing Materials

Polyimide (PI) is an organic polymer material that exhibits an outstanding comprehensive performance. It can operate continuously within a temperature range of −200 °C to 300 °C and possesses excellent wear and deformation resistance, making it a primary candidate material for high-speed ball bearing cages [15]. In addition, molybdenum disulfide (MoS_2_), with its typical two-dimensional layered structure, is widely employed as a lubricating filler in composite materials [16,17]. Both the thermoplastic polyimide (TPI, EXTEM™ XH1015, Sabic, Riyadh, Saudi Arabia) and the MoS_2_ nanoparticles (50–60 nm in diameter, Aladdin Co., Ltd., Shanghai, China) used in this study were commercially available. Based on previous findings that PI composites exhibit optimal tribological performance at a 15 wt% MoS_2_ content, this ratio was adopted for the present work [18].

**Figure 1:**
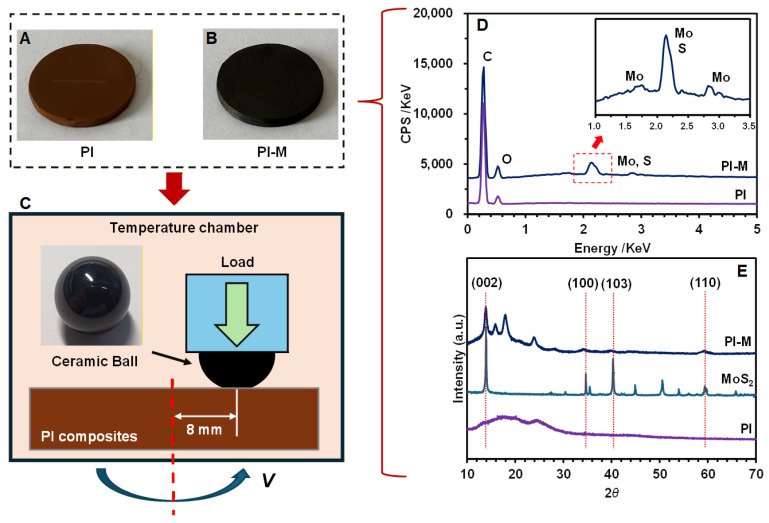
A schematic of the testing materials and methods: the manufactured pure PI (**A**) and PI-MoS_2_ (**B**) composite disks and the ball-on-disk test configuration within the temperature chamber (**C**). Energy-dispersive spectroscopy (EDS) (**D**) and X-ray diffraction (XRD) (**E**) were used to characterize the composites.

To ensure the uniform dispersion of MoS_2_ nanoparticles within the PI matrix, the two components were first thoroughly blended using a parallel twin-screw extruder (ZC-65D, Nanjing Zhicheng Rubber Machinery Co., Ltd., Nanjing, China). The resulting compound was pelletized and then injection molded into disk-shaped samples (20 mm × 2 mm) using an injection molding machine (DQ-180T, Quande Machinery Co., Ltd., Wuxi, China). The processing parameters were set as follows: a barrel temperature of 330 °C, a mold temperature of 200 °C, an injection pressure of 80 MPa, and a cooling time of 5 min. The obtained pure PI and PI-MoS_2_ (denoted as PI-M) samples are shown in Figure 1A and Figure 1B respectively.

The distribution of MoS_2_ in the composites was examined using energy-dispersive spectroscopy (EDS) and X-ray diffraction (XRD). An EDS analysis confirmed the presence of Mo and S elements in the PI-M composite, as indicated by the corresponding characteristic peaks in the spectrum (Figure 1D). Furthermore, XRD patterns of the PI-M composite displayed four distinct diffraction peaks corresponding to the (002), (100), (103), and (110) crystal planes of MoS_2_ (Figure 1E) [19]. These results collectively verify the successful incorporation and dispersion of MoS_2_ nanoparticles within the PI composite.

### 2.2. The Friction and Wear Tests Under High Temperatures

To mitigate the increasingly prominent issue of electrical discharge damage in drive motor bearings of EVs, insulating bearing solutions such as ceramic balls are being adopted in electric drive systems to replace conventional steel balls [20,21]. In this study, 10 mm diameter silicon nitride (Si3N4) ceramic balls, with a hardness of 77 HRC (Rockwell C scale), were applied. Accordingly, in this study, ceramic balls and PI composites were selected as the friction pair. Tests were conducted on a multifunctional standard tribometer (Jinan Yihua Tribological Testing Technology Co., Ltd., Jinan, China). During the operation of a ball bearing, the cage remains relatively fixed while the rotating balls slide against it. Therefore, a ball-on-disk rotating sliding configuration was employed. As illustrated in Figure 1C, the ceramic ball was fixed at the upper station, while the PI composite disk was rotated by a motor at the lower station under dry friction conditions.

The normal load was set to 20 N, and the sliding speed was maintained at 0.21 m/s. All experiments were performed inside a sealed temperature chamber at five controlled temperatures: 15 °C, 50 °C, 100 °C, 150 °C, and 200 °C. Prior to each test, both the specimen and the chamber were stabilized at the target temperature for 1 h. And each test lasted 30 min, during which the friction force and coefficient were recorded online at a sampling frequency of 500 Hz. The friction and wear tests were conducted in an ambient atmosphere (relative humidity: 45 ± 5%). In addition, all tests were repeated three times.

Before testing, the surfaces of the composite samples and the ceramic ball were polished with 2000-grit wet sandpaper to achieve an average surface roughness (Sa) of 0.04 ± 0.01 μm and 0.01 ± 0.01 μm individually, as measured by a white-light interferometer (Micro Xam, ADEP Phase Shift, Inc., Tucson, AZ, USA). And both the composite disks and ceramic balls were ultrasonically cleaned in anhydrous ethanol for 10 min and then dried with warm air to remove any surface contaminants.

### 2.3. Measurement Techniques and Equipment

The wear surface topography of the composite samples was measured using a laser confocal microscope (VK-X1000, KEYENCE, Osaka, Japan). The microscopic wear morphology was examined with scanning electron microscopy (SEM, VEGA3, TESCAN, Brno, Czech Republic). The viscoelastic properties of the two composites were characterized by a dynamic mechanical analysis (DMA, DMA850, TA Instruments, Newcastle, WA, USA). Furthermore, the pyrolysis characteristics of the composites were investigated through a thermogravimetric analysis (TGA, TGA2 thermogravimetric analyzer, Mettler Toledo, Columbus, OH, USA, interfaced with an IS50 FTIR spectrometer, Thermo Fisher Scientific, Waltham, MA, USA).

## 3. Results

### 3.1. The Coefficient of Friction (COF) of Two Composites Under Different Temperatures

**Figure 2:**
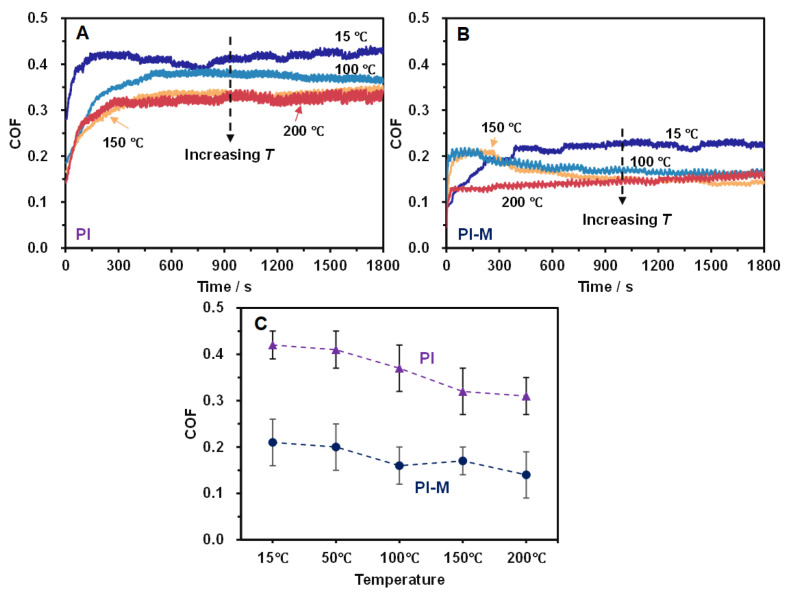
The COF curves of the pure PI (**A**) and PI-M (**B**) under the increasing temperatures as well as their average COF results (**C**).

Figure 2 presents the COF curves of PI and PI-M composites over 30 min under a load of 20 N, a sliding speed of 0.21 m/s, and different temperature conditions. The two composites exhibited distinct friction responses with increasing ambient temperatures. As shown in Figure 2A, the COF of the pure PI initially rose and then stabilized around 0.4 at 15 °C. When the temperature increased to 100 °C, the coefficient still followed a similar rising-then-stabilizing trend but decreased to approximately 0.35. At 150 °C and 200 °C, the PI’s COF remained steady at about 0.32 and 0.31, respectively, indicating a gradual reduction in the COF with the temperature. Figure 2B demonstrates that under the same test conditions, the COF of the PI-M composite was notably lower than that of the pure PI. At 15 °C, the coefficient rose initially and stabilized near 0.21 after 450 s. As the temperature increased, the COF also declined: it dropped below 0.20 at 100 °C, stabilized around 0.15 at 150 °C, and reached its lowest steady value near 0.13 at 200 °C.

The averaged COF was calculated from all friction data recorded during the steady state period, and results are shown in Figure 2C. At 15 °C, the average COF of PI was 0.42, while that of PI-M was 0.21. As the temperature increased, the coefficients of PI were measured as 0.41 (50 °C), 0.36 (100 °C), 0.32 (150 °C) and 0.31 (200 °C). In contrast, the corresponding values for PI-MoS_2_ were 0.21 (50 °C), 0.16 (100 °C), 0.17 (150 °C) and 0.15 (200 °C). These results indicated that the addition of MoS reduced the average COF of the composite by approximately 50% compared with the pure PI.

Moreover, the friction behavior of polymer materials is governed mainly by adhesive forces at the contact interface, the hysteresis component due to the strain lag behind stress, and the plowing effect [22]. With rising temperatures, the frictional heating increased rapidly at the contact surface, which facilitated shear between molecular chains and promoted segmental motion along the sliding direction. This process reduced adhesive forces, thereby lowering the COF [23]. Consequently, both PI and PI-MoS_2_ exhibited a decreasing trend in friction coefficients as the temperature increased.

### 3.2. The Wear Profile and Wear Volume of Two Composites at Different Temperatures

**Figure 3:**
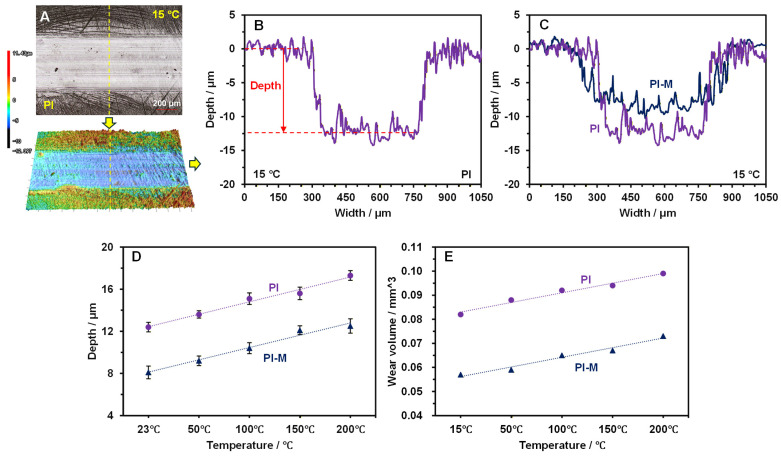
The 3D morphology (**A**) and contour (**B**) of the wear marks on the surface of PI at 15 °C, as well as the comparison result with the PI-M (**C**); (**D**,**E**) show the wear depth and wear volume results of two materials at different temperatures.

The wear properties of PI and PI-M were evaluated under a load of 20 N, a sliding speed of 0.21 m/s, and a duration of 30 min at different temperatures, and results are displayed in Figure 3. Wear scar profiles and 3D topographies were characterized using laser confocal microscopy (LCSM). As shown in Figure 3A,B, the wear scar morphology and its profile curve for the pure PI at 15 °C exhibited a maximum depth of 12.3 μm. However, the application of MoS_2_ reduced the wear depth to 8.1 μm at the same temperature (Figure 3C). Figure 3D illustrates that the wear depth of both composites increased with the temperatures. For PI, the depths were 13.6 μm (50 °C), 15.1 μm (100 °C), 15.6 μm (150 °C) and 17.3 μm (200 °C). Under the same conditions, PI-M exhibited depths of 9.2 μm, 10.4 μm, 12.1 μm, and 12.5 μm, representing a reduction of approximately 20% compared to the pure PI.

The wear volume was calculated by applying the integral principle to the measured cross-sectional curves. *V* represents the wear volume, and it was determined by the wear distance *L*, the wear width *W*, the unit increment length (unit testing interval) ∆x, and the wear depth of the *i*th rectangle (*f* (*x_i_*)). The value of *L* (sliding distance) is the sliding distance during the ball-on-disk friction and wear test, which is calculated as the circumference of the circular wear track:

*L* = 2π*r*

*r* is the radius of the wear track (the distance from the center of the disk to the contact point of the ceramic ball), which is 8 mm in our experimental setup.

With ∆x having a testing interval of 0.001 mm, any area (*S_ai_*) of the unit rectangle can be calculated as

$$S_{ai}=f(x_{i})∆x$$

The full cross-section of the wear scratch thus had an area of


$$S_{all}=\sum_{i=1}^{W/∆x}S_{ai}$$

Therefore, the whole wear volume could be calculated as

$$V=S_{all}\times L=L\times \sum_{i=1}^{W/∆x}f(x_{i})∆x$$

The calculated wear volumes under different temperatures are shown in Figure 3E. At 15 °C, the wear volume of the PI was 0.082 mm^3^, while that of PI-M was 0.057 mm^3^. With increasing temperatures, the wear volume of the PI rose to 0.088 (50 °C), 0.092 (100 °C), 0.094 (150 °C) and 0.099 (200 °C) mm^3^. In contrast, the addition of MoS_2_ reduced the wear volume to 0.059 (50 °C), 0.065 (100 °C), 0.067 (150 °C) and 0.073 (200 °C) mm^3^. These results demonstrated that PI-M possesses a superior high-temperature wear resistance compared to the pure PI.

### 3.3. The Contact Deformation Behaviors of Two Composites at Different Temperatures

**Figure 4:**
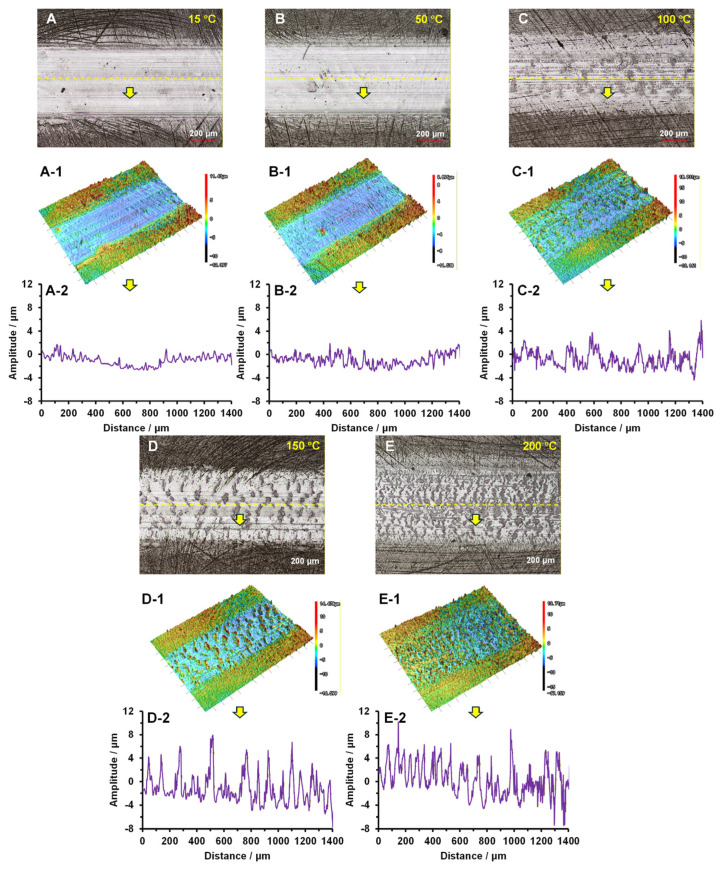
The contact deformation behaviors of PI at different temperatures: (**A**–(**A-2**)) 15 °C; (**B**–(**B-2**)) 50 °C; (**C**–(**C-2**)) 100 °C; (**D**–(**D-2**)) 150 °C; and (**E**–(**E-2**)) 200 °C.

Figure 4 presents the surface contact deformation behavior of the PI under different temperatures after testing at a speed of 0.21 m/s, a load of 20 N and a duration of 30 min. The 3D morphology and deformation profile data of the contact deformation were obtained using Laser Confocal Scanning Microscopy (LCSM). At 15 °C, the optical results (Figure 4A) and the 3D topography (Figure 4(A-1)) revealed almost no contact deformation on the worn surface, with the average amplitude of the deforming fluctuation ranging from −1 to 1.1 μm, as shown in Figure 4(A-2). At 50 °C, slight contact deformation was observed on the worn surface, and the fluctuation amplitude increased to the range of −1.7 to 2.2 μm, as illustrated in Figure 4B–(B-2). At 100 °C, prominent contact deformation occurred on the PI surface, characterized by a fish scale pattern of deformation along the sliding direction with corresponding fluctuations (Figure 4C,(C-1)), which periodically varied between −3.8 and 4.1 μm (Figure 4(C-2)). The fish-scale-like deformation became more severe with increasing temperatures. At 150 °C, extensive periodic deformation behavior with fluctuation amplitudes between −4.1 and 7.7 μm was observed, while at 200 °C, such deformation became even more densely distributed across the entire wear track, as proven in Figure 4D–(D-2),E–(E-2).

**Figure 5:**
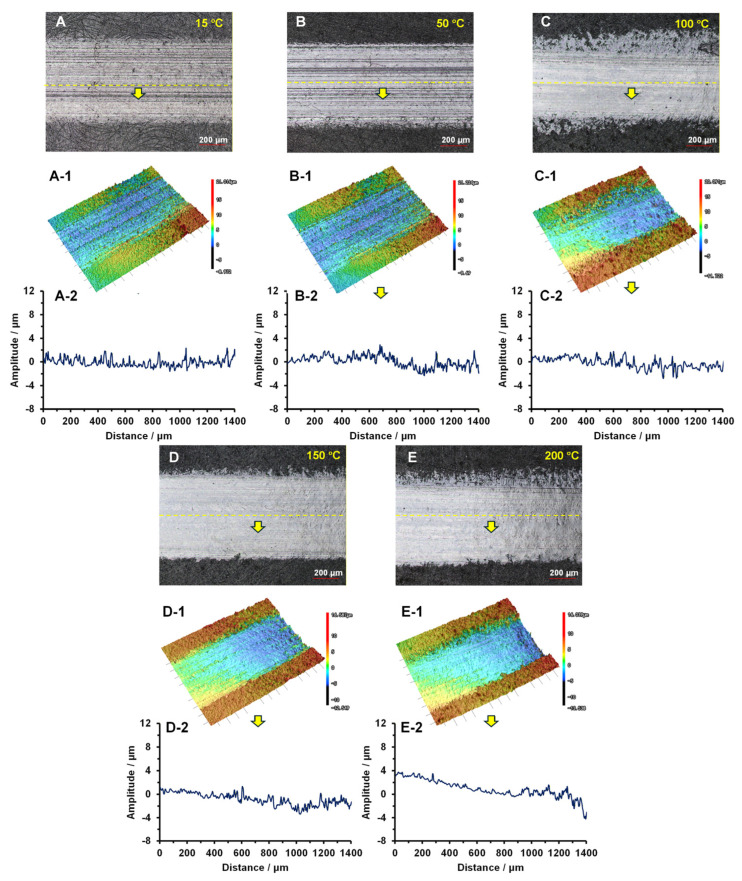
The contact deformation behaviors of PI-M at different temperatures: (**A**–(**A-2**)) 15 °C; (**B**–(**B-2**)) 50 °C; (**C**–(**C-2**)) 100 °C; (**D**–(**D-2**)) 150 °C; and (**E**–(**E-2**)) 200 °C.

Figure 5 presents the 3D morphology and corresponding profile of the surface contact deformation for PI-M under the same test conditions. At 15 °C and 50 °C, the worn surface was dominated by groove deformation along the rolling friction direction, with small deformation amplitudes fluctuating within the range of 0 to μm, as shown in Figure 5A–(A-2),B–(B-2). As the temperature rose to 100 °C, the groove deformation gradually diminished, and the fluctuation range of the deformation amplitude increased from −0.5 to 1.2 μm, as illustrated in Figure 5C–(C-2). And the worn surface remained relatively smooth without apparent contact deformation at 150 °C and 200 °C, while the surface deformation amplitude almost remained around 0 μm (as proven in Figure 5D–(D-2),E–(E-2)). These results indicated that MoS_2_ effectively reduced the surface contact deformation behavior of the PI under high-temperature conditions and enhanced its resistance to deformation.

### 3.4. Microstructure of Worn Surfaces of These Two Composites at Different Temperatures

**Figure 6:**
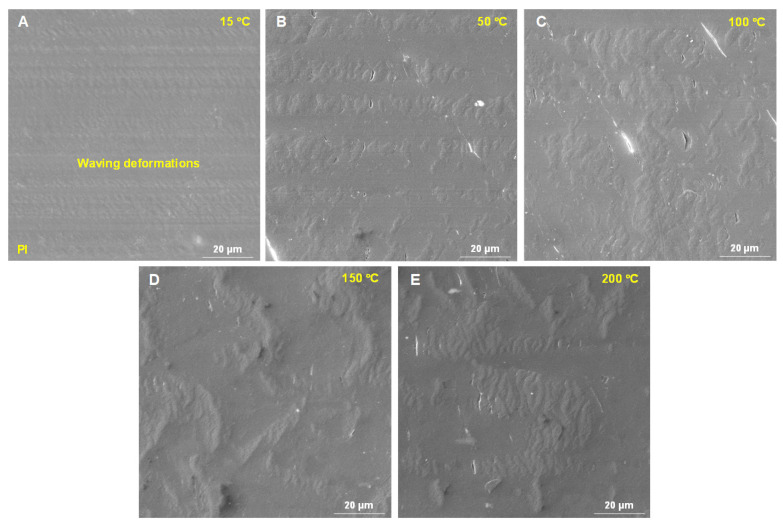
The microstructure of the PI’s worn surface at different temperatures: (**A**) 15 °C; (**B**) 50 °C; (**C**) 100 °C; (**D**) 150 °C; and (**E**) 200 °C.

To further investigate the tribological mechanisms of PI, the surface microstructures after friction and wear tests under different temperature conditions were examined, as shown in Figure 6. At 15 °C, slight and periodically occurring contact deformation was observed within the worn track of PI (Figure 6A). As the temperature increased to 50 °C, the periodic contact deformation became more pronounced, exhibiting a fish scale pattern (Figure 6B). At 100 °C, the elevated temperature induced adhesive wear on the contact surface, enlarging the fish scale deformation (Figure 6C). This could be attributed to the combined effect of the ambient temperature and frictional heating, which raised the temperature at the friction interface and made the polymer more prone to extrusion and deformation [24]. Consequently, the worn surface exhibited pronounced thermoplastic deformation, cracking, stretching, tearing, and material agglomeration [25]. Therefore, at 150 °C and 200 °C, the fish-scale-like deformation was intensified (Figure 6D,E). This corroborated that the morphological fluctuations observed in Figure 4 originated from periodic contact deformation during the friction and wear process.

**Figure 7:**
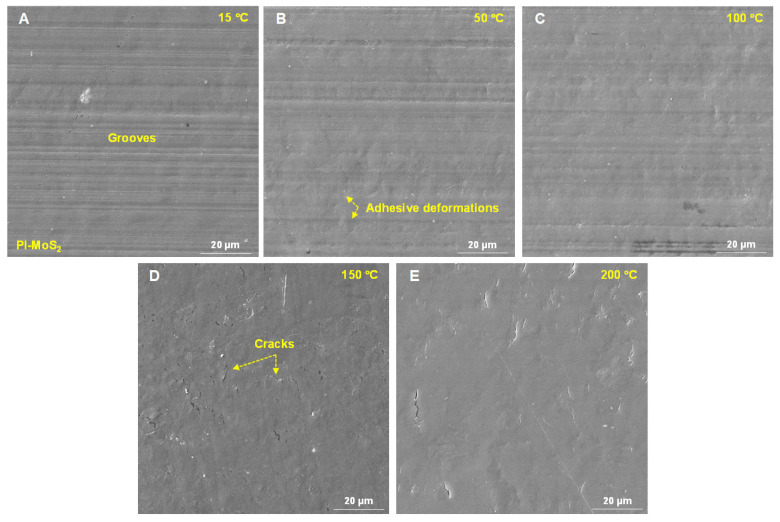
The microstructure of PI-M’s worn surface at different temperatures: (**A**) 15 °C; (**B**) 50 °C; (**C**) 100 °C; (**D**) 150 °C; and (**E**) 200 °C.

Figure 7 presents the microstructures of the PI-M worn surface after the testing under different temperatures. The analysis of these micrographs revealed that the composite surface did not exhibit the distinct fish-scale-like contact deformation behavior typically observed in the unreinforced PI. At 15 °C, the primary wear feature consisted of shallow, directional grooves aligned parallel to the sliding direction, indicative of mild abrasive or plowing mechanisms, as shown in Figure 7A. By increasing the temperature to 50 °C and 100 °C, the prominence of these grooves gradually diminished, and the adhesive interaction caused the local surface to roughen, as illustrated in Figure 7B,C. However, even at elevated temperatures of 150 °C and 200 °C, the worn surface retained a relatively smooth topography. While some fine, thermally induced micro-cracks were observed, large-scale plastic deformation or pronounced periodic patterning was effectively suppressed, as seen in Figure 7D,E. This sustained surface integrity and absence of severe morphological deformation across high temperatures were attributed to the solid lubricating function of the MoS_2_. The results clearly demonstrate that the ability of the PI-M composite to maintain a stable and smooth contact surface under varying thermal loads was a fundamental factor underpinning its consistently low and stable COFs and surface deformation fluctuations.

## 4. Discussion

**Figure 8:**
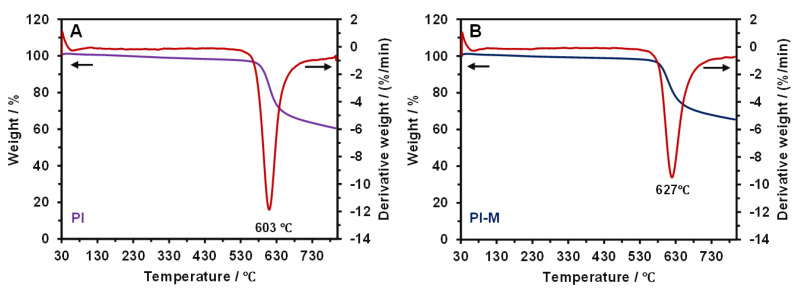
Thermogravimetric analysis results for PI (**A**) and PI-M (**B**).

Figure 8 presents the derivative thermogravimetry (DTG) curves of the pure PI and the PI-MoS_2_ composite, respectively. The DTG peak for the pure PI, corresponding to its maximum rate of mass loss, occurred at 603 °C (Figure 8A). At this temperature, the scission of molecular chains within the polymer led to irreversible alterations in the material’s structure and properties, ultimately resulting in material failure [26]. In contrast, the maximum mass loss rate for the PI-M was observed at a higher temperature of 627 °C, which indicated enhanced thermal stability due to the incorporation of MoS_2_. This was because MoS_2_ restricted the polymer chain segment mobility and increased the energy required for chain scission [27]. As a result, this shift in the decomposition temperature demonstrated that the composite retained structural integrity under more severe thermal conditions. Consequently, during friction and wear processes, the PI-M composite exhibited a superior resistance to the frictional heat generated at the sliding interface, contributing to its improved tribological performance at elevated temperatures.

**Figure 9:**
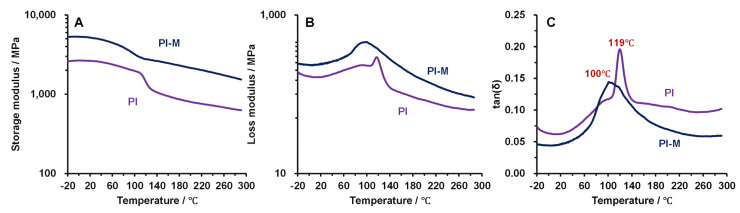
DMA results for PI and PI-M respectively: (**A**) storage modulus; (**B**) loss modulus; and (**C**) loss factor.

Figure 9 presents the dynamic mechanical analysis (DMA) results of the two composites, starting from −20 °C to fully and accurately characterize the viscoelastic relaxation transition of the composite materials. As the temperature increased, the storage modulus of both composites exhibited a declining trend. However, the storage modulus of the PI-M remained substantially higher than that of the pure PI throughout the tested range, as shown in Figure 9A. This indicated that the molecular chains within the composite possessed a greater capacity for storing reversible deformation energy, reflecting enhanced elasticity and rigidity [28]. Conversely, the loss modulus of PI-M exceeded that of the PI over the entire temperature range (Figure 9B), which suggested that the PI-M exhibited more pronounced viscous behavior and a higher damping capacity [29]. Figure 9C shows that PI-M displayed a lower loss factor than the PI. The low loss factor (tan δ) of a polymer material signified weak viscoelasticity, allowing molecular chain segments to move in sync with stress changes so that the mechanical energy was primarily stored as elastic potential rather than being dissipated as heat [30]. Consequently, during friction and wear, surface contacts predominantly induced recoverable elastic deformation instead of significant plastic flow or viscous dissipation, resulting in minimal surface deformation, as proven in Figure 5 and Figure 7.

In addition, the onset of fish-scale-like contact deformation in the pure PI at temperatures above 100 °C correlates well with the decline in the storage modulus observed in the DMA, which marks the vicinity of its glass transition temperature (Tg). This transition facilitates molecular chain mobility and reduces the material’s ability to resist cyclic frictional stress, thereby promoting periodic plastic deformation. The incorporation of MoS2 shifts this behavior to higher temperatures by restricting chain motion and improving thermal stability, as reflected in both the DMA and TGA results. 

**Figure 10:**
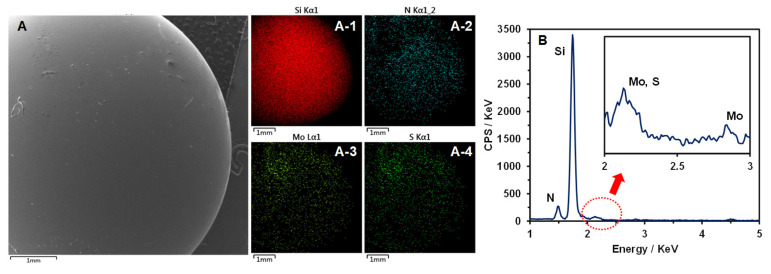
Results of the element distribution (**A**–(**A-4**)) and curve (**B**) on the surface of the ceramic ball after friction with PI-M at 100 °C.

However, in the temperature range of 82–113 °C, the loss factor of PI-M was lower than that of the pure PI (Figure 9C), indicating relatively weaker elastic properties of the composite within this specific interval. Nevertheless, the elemental analysis performed on the surface of the ceramic ball used against PI-MoS_2_ under a 100 °C testing condition confirmed the presence of both Mo and S on the ball surface, with a uniform distribution observed (Figure 10A–(A-4)). Furthermore, characteristic peaks for these elements were clearly identified in the corresponding EDS spectrum (Figure 10B). These findings demonstrated that during the friction and wear process, the MoS_2_ within the composite exuded onto the contact interface, forming a continuous lubricating film that enhanced the interfacial lubrication. Consequently, even though the elastic performance of PI-M was compromised within this temperature range, the lubricating film present at the interface effectively reduced the surface deformation behavior by diminishing the cutting and plowing forces during sliding contact.

## 5. Conclusions

This study investigated the tribological properties of PI and PI-M composites under elevated temperature conditions. The main conclusions are summarized as follows:

(1). Compared with the pure PI, the PI-M composite exhibited a 50% reduction in the friction coefficient (COF) and around a 30% decrease in wear volume;

(2). The incorporation of MoS_2_ enhanced the thermal stability of the composite, effectively suppressing the contact deformation caused by the combined effect of frictional heating and mechanical stress during dry sliding against a ceramic ball;

(3). The MoS_2_ modification lowered the loss factor of the PI, improved the elastic response of the composite, and consequently eliminated the fish-scale-like contact deformation at the worn surface;

(4). During the friction process, MoS_2_ was progressively transferred to the contact surface, forming a lubricating film to improve the interfacial lubrication across various temperatures.

## Data Availability

The data presented in this study are available on request from the corresponding author.
